# Prediction during statistical learning, and implications for the implicit/explicit divide

**DOI:** 10.2478/v10053-008-0115-z

**Published:** 2012-05-21

**Authors:** Rick Dale, Nicholas D. Duran, J. Ryan Morehead

**Affiliations:** 1 Cognitive and Information Sciences, University of California, Merced, USA; 2 Department of Psychology, University of California, Berkeley, USA

**Keywords:** prediction, consciousness, dynamics, implicit learning, statistical learning, serial reaction time, computer-mouse tracking

## Abstract

Accounts of statistical learning, both implicit and explicit, often invoke predictive processes as central to learning, yet practically all experiments employ non-predictive measures during training. We argue that the common theoretical assumption of anticipation and prediction needs clearer, more direct evidence for it during learning. We offer a novel experimental context to explore prediction, and report results from a simple sequential learning task designed to promote predictive behaviors in participants as they responded to a short sequence of simple stimulus events. Predictive tendencies in participants were measured using their computer mouse, the trajectories of which served as a means of tapping into predictive behavior while participants were exposed to very short and simple sequences of events. A total of 143 participants were randomly assigned to stimulus sequences along a continuum of regularity. Analysis of computer-mouse trajectories revealed that (a) participants almost always anticipate events in some manner, (b) participants exhibit two stable patterns of behavior, either reacting to vs. predicting future events, (c) the extent to which participants predict relates to performance on a recall test, and (d) explicit reports of perceiving patterns in the brief sequence correlates with extent of prediction. We end with a discussion of implicit and explicit statistical learning and of the role prediction may play in both kinds of learning.

## Introduction

To what extent is prediction related to sequential learning and memory, and to implicit or explicit knowledge of that learning? In this paper, we offer a novel methodology that may help answer this question, and present experimental results that suggest this methodology holds promise for connecting these phenomena: prediction, statistical learning, and explicit awareness. In brief, our experiment is a simple manual spatial-position tracking task, in which a participant’s behavior is tracked with the computer-mouse cursor. We are thus able to detect predictive movements readily. We show that predictive behaviors emerge quickly in a simple short-sequence design, using 48-element sequences of varying grammatical regularity. Prediction, learning, and explicit knowledge all correlate strongly.

Many researchers in diverse domains of cognitive science have identified prediction as central to perception, cognition, and action (Bar, 2009; Bieri, 1955; Bubic, von Cramon, & Schubotz, 2010; Cleeremans & McClelland, 1991; Craik, 1943; Elman, 1990; Enns & Lleras, 2008; Kveraga, Ghuman, & Bar, 2007; Neisser, 1976; Ramscar, Yarlett, Dye, Denny, & Thorpe, 2010; Rao & Sejnowski, 2003; Reynolds, Zacks, & Braver, 2007; Schubotz, 2007; Shelhamer & Joiner, 2003; Sutton & Barto, 1998; Wolpert & Flanagan, 2001). This has been especially true of theories of statistical learning (Cleeremans & McClelland, 1991; Hoffmann, Martin, & Schilling, 2003; Hunt & Aslin, 2001; Nissen & Bullemer, 1987; Stadler, 1989). As we argue below, these theories are almost always based on indirect evidence for prediction: Virtually all statistical learning and serial reaction time (SRT) experiments employ reaction-time (RT) methods that do not reveal predictive behaviors *during* learning (D. J. Marcus, Karatekin, & Markiewicz, 2006). Yet such experiments cannot mediate between predictive, forward-looking theories of statistical learning processes (Cleeremans & McClelland, 1991; Nissen & Bullemer, 1987), and theories that could be based more on associative memory traces that link event to event, and need not invoke forward-looking learning processes (e.g., Jones & Pashler, 2007). The former family of theories needs a more direct technique to tap prediction and to support this common assumption.

In this paper, we develop a technique for capturing prediction behavior directly, and present a simple experimental demonstration of it that speaks to this theoretical aforementioned issue. Our results show that even if a brief sequential pattern is sufficiently ordered, participants begin to actively predict what stimulus will follow from a previous one, revealing prediction as a wholesale, active strategy. Participants who do so more also tend to report more explicit awareness of pattern in that stimulus sequence. Our data suggest that indeed prediction is central to learning and awareness, and may reveal how *different* forms of prediction may correlate with implicit or explicit awareness, which themselves are dependent upon the relative transparency of the structure being learned. In what follows, we first briefly review theoretical issues, and argue that predictive versus associative mechanisms can easily be confounded in statistical learning experiments. We then present our experimental results, and discuss their theoretical implications in the general discussion.

### Predictive versus associative processes for learning

Distinguishing between predictive and associative learning mechanisms is a problem that extends across many domains in cognitive psychology, given that “...efficient processing of events in ambiguous contexts does not need to result from effective preparation, but retrospective use of information regarding events which occurred following those of interest” (Bubic et al., 2010, p. 2). Some studies have sought to tease apart these two potential mechanisms, predictive versus retrospective processing, in behavioral experiments. For example, Enns and Lleras (2008) showed that visual search from a recent scene in memory can be so fast (“rapid resumption”) that only a predictive mechanism could sensibly explain their findings. They noted that this is in contrast to fluid access to recent memory, characteristic of more retrospective processes. These same issues are faced by RT facilitation findings in sequential tasks that reveal statistical learning.

Consider the findings of Hunt and Aslin (2001), whose study is similar to the design we employed here. They evaluated movement speed to predictable stimuli within a visuospatial layout. In their experiment, the stimuli corresponded to seven lights that were arranged equidistantly in a semicircle. Participants were instructed to press the lights and then return to a fixed position below the lights to trigger the next position. They showed learning of *predictable* positions across trials of the experiment, finding that RTs were fastest for stimuli in predictable positions.

Though Hunt and Aslin (2001) were primarily interested in the type of cues that underlie learning in this serial-reaction design, the cognitive process that was assumed to be operating in this context was one of an anticipatory nature: “If it [RT] was faster, this implied that the participant was relatively more certain about the subsequent element in the sequence of stimuli and was able to anticipate the correct transition and produce a faster response” (p. 670). One issue with this general assumption is that faster latencies may simply reflect a capacity to react more quickly given the strength of local “memory traces” induced during learning. As another example, in a recent computational model of SRT learning, Jamieson and Mewhort (2009) require only very local memory cuing, and the model by itself is not equipped with a predictive mechanism. It is in fact retrospective (in the sense of Bubic et al., 2010) because the generation of any new response is carried out through integrating information from the previous stimulus and response, and with this simple local process, it can capture a wide range of basic statistical-learning results. A forward-looking model would produce expectations or anticipations for the next stimulus. This is an important distinction that should not be trivialized. The relevance of a current stimulus is evaluated *when it is seen* in a retrospective model, rather than *before it is seen*, which is how a predictive, anticipatory process would function. So a memory-based retrospective process could facilitate these responses in some manner, without being explicitly predictive. In short, the current stimulus is simply faster to process when it is seen.

The distinction between predictive and retrospective processes can be strengthened by considering two ways in which computational models could capture response facilitation. In a sequential statistical learning experiment, where we attempt to model the processing of some stimulus S_N_ that has followed from some previous set of stimuli (S_N-1_, S_N-2_, etc.), there are two ways in which speeded processing of S_N_ might happen. A retrospective model would process this S_N_ more quickly if its (activated) memory traces for preceding material facilitate that processing. No preparation or prediction is required for this to be so: Any of various processes required for evaluating the new S_N_ could be facilitated by having relevant recent representations active before S_N_. A predictive model, however, does not wait to be facilitated in this processing. Instead, it would in some manner or another have at least part of the requisite processing of S_N_ already in place even before S_N_ appears, because seeing S_N-1_, S_N-2_, etc. together encourages the system to “look forward” towards upcoming stimulus events. There is a computational precedent for this distinction. In the retrospective case, McClelland (1979) introduced a cascade algorithm to model facilitated processing time in an associative feedforward network. In the predictive case, the well-known simple-recurrent network architecture has been used to model SRT and statistical learning data (Cleeremans & McClelland, 1991; Misyak, Christiansen, & Tomblin, 2009). Yet neither of these processes, prima facie, is fundamentally or a priori the most desirable. We would agree that statistical learning studies offer considerable evidence that anticipation is pervasive. Despite this pervasiveness, some recent works suggest that this pervasiveness should not be taken for granted in any given situation, and that direct evidence for such a central mechanism, and its benefits, ought to be obtained (e.g., Jones & Pashler, 2007).

The vast majority of research on statistical learning has employed indirect measures of anticipation. Reber (1967) used grammatical endorsement scores in test phases as evidence of learning. Looking time has been used in statistical learning studies with infants and young children (see Saffran, 2003, for a review). SRT studies virtually all use basic RT measures in training phases, from Nissen and Bullemer (1987) onward. There are a few studies that reveal anticipatory tendencies, such as those that show the emergence of predictive errors in SRT studies (e.g., Schvaneveldt & Gomez, 1998), yet these predictive errors may still be evidence of some learning having already taken place. In other words, prediction may not have been part of the learning process, but rather an effect. There is indeed some good evidence that ocular and manual movements display predictive behaviors during statistical learning and that this prediction relates to explicit knowledge. D. J. Marcus et al. (2006) showed that eye-movement anticipations are frequent in an SRT task, and relate to learning and explicit knowledge. Duran and Dale (2009) showed some weak evidence for anticipatory manual responses in a statistical learning paradigm similar to Saffran, Aslin, and Newport (1996; see also Moisello et al., 2009). The current experiment complements these findings and supplies a potential experimental framework for systematic exploration of prediction, and possibly, different types of prediction strategies. For example, here we show that manual anticipation can take on two different forms: “optimized” reaction (where one readies the response but does not try to make it beforehand), and explicit “wagers,” which appear to accompany explicit learning, in which a participant actually heads for the next expected stimulus prior to its appearance. In our experiment, we observe that participants first find an optimal cursor location from which to react while waiting for another stimulus to appear (without predicting a specific stimulus), though at some point during the experiment they become aware of a pattern and begin to explicitly predict or “wager” its next occurrence. Once they do predict, it becomes a stable strategy that guides learning of a short sequence.

In summary, most statistical learning experiments, whether explicit or implicit in their learning outcomes, are based on indirect information about anticipatory processes consistent with both anticipatory and non-anticipatory types of models. We would agree with many researchers who have argued that anticipation seems to be the most appealing cognitive process for handling events in time (as cited above, and as discussed in the implicit learning literature, e.g., in D. J. Marcus et al., 2006; Nissen & Bullemer, 1987; Schvaneveldt & Gomez, 1998; Stadler, 1989). But obtaining direct evidence of this process, and developing an empirical framework to further explore prediction in statistical learning, would further help to connect this mechanism for learning *during learning*.

In this paper, we are not arguing that implicit learning is either purely associative or predictive, for it may be that they both work together during learning (and indeed, predictions may use associative mechanisms at root; e.g., Bar, 2009). Our point is that theories that use concepts of prediction could be further supported by directly revealing prediction in behavior. The purpose of this paper is to showcase an experimental paradigm that can reveal predictive processes, and to explore the properties of this anticipation/prediction as it relates to memory for sequences and explicit knowledge of the regularity of these sequences. We indeed find rich patterns of prediction, and this prediction relates to learning and explicit awareness of that learning.

## Experiment

Here, we use an experimental design that reveals manual prediction, and we investigate the properties of this behavior. To provide an unambiguous measure of prediction, we turned to the measurement of hand movement during task performance. In several recent studies, the semi-continuous movements of the computer-mouse cursor were regarded as a direct and (occasionally) uninterrupted translation of unfolding cognitive processes (Song & Nakayama, 2009; Spivey, Grosjean, & Knoblich, 2005). Motivated by this logic, we tracked participants’ computer mouse as they clicked on a visual cue that moved around a spatial landscape on the computer screen. Every time the cue was clicked, it momentarily disappeared before reappearing in a new location. During this period of disappearance, the learner had an opportunity to predict the most likely region of reappearance of the cue (it is like a simplified version of the “Whac-A-Mole” classic arcade game that readers may be familiar with). By tracking the coordinates of the computer-mouse, results can show when (or if) participants manually gravitate towards predictable regions. We used this in a simple sequence-learning task, in the spirit of Nissen and Bullemer (1987) and of Hunt and Aslin (2001). The task requires participants to respond to spatial stimuli that occur in sequences that vary in their ordering regularities.

### Methods

#### Participants

We recruited 143 participants from Amazon Mechanical Turk (www.mturk.com). This system has, in several previous studies, produced extremely reliable respondents even in relatively cognitive-intensive tasks such as data coding (Kittur, Chi, & Suh, 2008; Snow, O’Connor, Jurafsky, & Ng, 2008; Sorokin & Forsyth, 2008). Our participants were compensated with a small monetary reward for the task, which required approximately 10-15 min.

#### Stimuli and interface

**Figure 1.**
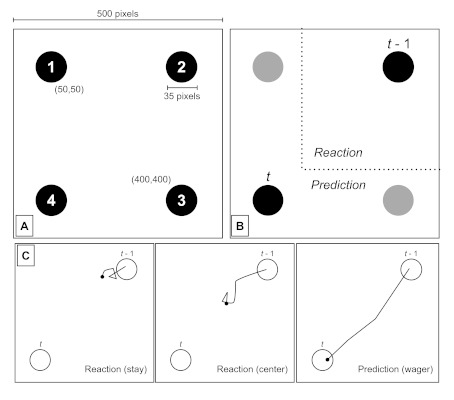
Panel A. Stimuli locations on computer screen. Panel B. Example region for reactive and predictive movements when mouse position is recorded after disappearance of stimulus *t*-1 and at the onset of stimulus *t* (750 ms lapsed). Panel C. Hypothetical trajectory examples for illustration of predictive/reactive categories during the 750-ms inter-stimulus interval. (Note that open circles reflect no stimuli on the screen, but indicate stimulus *t* and *t*-1.)

The interface was programmed using Adobe Flash, in which the computer-mouse cursor could be accessed for its x and y coordinates at a rate of approximately 40 Hz. The interface occupied a 500-by-500-pixel region within the users’ Internet browser (see Figure 1, Panel A).Target stimuli were 35-pixel-diameter black circles that appeared individually during training, arrayed in a 2 × 2 grid. Participants used only their computer pointer to interact with the interface.

We constructed 11 training sequences of 48-circle appearances using the constraint that a circle in one position could not appear consecutively. In addition, each position (1-4) appeared an equal number of times. Each sequence contained an order of circle positions of varying regularity. From Jamieson and Mewhort (2009), *regularity* (*G*) was defined as the extent to which a 48-position sequence is redundant. *G* lies between 0 (no regularity) and 1 (high regularity), and we chose an array of values of *G* for our 11 sequences, including three sequences with perfect regularity, with the aim of having highly regular sequences in which prediction would be observed. Table 1. details this redundancy statistic, the stimulus sequences, and the regularity of each. Sequences of growing regularity have emerging patterning of the four positions, with fewer and fewer irregular trials intervening between these patterns.

**Table 1.** The Stimulus Sequences by Circle Position

*n*	Sequence	*G*
11	4-2-3-2-1-2-1-2-4-1-3-2-1-4-1-3-1-3-2-4-1-3-1-3-1-4-2-4-3-4-2-4-1-3-1-4-2-3-2-4-1-4-2-3-4-3-2-3	.25
14	1-2-3-1-3-1-2-1-2-1-3-4-3-4-1-2-4-2-4-2-4-3-1-2-4-3-4-2-4-3-4-3-1-2-3-4-2-1-3-4-2-1-3-1-3-4-2-1	.40
16	2-1-4-3-4-3-2-4-1-2-3-2-1-2-1-4-3-4-3-2-3-2-3-2-1-3-2-1-4-1-4-1-4-3-4-1-2-1-4-1-4-3-2-3-2-3-4-1	.44
11	4-2-3-4-2-1-3-1-3-1-3-1-3-4-2-4-2-4-2-1-3-1-3-1-3-4-2-3-4-2-1-3-1-3-4-1-2-1-2-4-2-4-2-1-3-4-2-4	.55
11	4-3-4-3-4-3-2-1-4-3-2-1-4-3-2-1-4-3-2-1-4-3-2-1-3-2-1-4-3-2-1-3-2-1-2-1-4-3-2-1-2-1-4-3-4-2-1-4	.68
13	3-2-3-2-4-1-3-2-4-1-4-1-3-2-3-2-3-2-4-1-3-2-4-1-3-2-3-2-4-1-3-2-4-1-4-1-3-2-4-1-4-1-4-1-4-1-3-2	.76
10	2-3-1-4-1-4-1-4-2-3-1-4-2-3-1-4-2-3-1-4-2-3-1-4-2-3-2-3-2-3-2-3-1-4-1-4-2-3-1-4-2-3-1-4-2-3-1-4	.79
15	2-3-4-1-2-3-4-1-2-3-2-3-4-1-2-3-4-1-2-3-4-1-2-3-4-1-2-3-4-1-4-1-2-3-4-1-2-3-4-1-2-3-4-1-2-3-4-1	.89
15	2-1-3-4-2-1-3-4-2-1-3-4-2-1-3-4-2-1-3-4-2-1-3-4-2-1-3-4-2-1-3-4-2-1-3-4-2-1-3-4-2-1-3-4-2-1-3-4	1,0
14	1-2-4-3-1-2-4-3-1-2-4-3-1-2-4-3-1-2-4-3-1-2-4-3-1-2-4-3-1-2-4-3-1-2-4-3-1-2-4-3-1-2-4-3-1-2-4-3	1,0
11	4-3-2-1-4-3-2-1-4-3-2-1-4-3-2-1-4-3-2-1-4-3-2-1-4-3-2-1-4-3-2-1-4-3-2-1-4-3-2-1-4-3-2-1-4-3-2-1	1,0

*Note*. Stimulus sequences were numbered as in Figure 1. *n* = number of subjects randomly assigned to this training sequence. *G* is a grammatical regularity score taken from Jamieson and Mewhort’s (2009) study of implicit learning. *G* = 1 – *U*(sequence)/*U*(random) where *U*(sequence) is equal to the first-order entropy of the sequence. This is simply equal to the entropy of the probability distribution of transitions from positions *i* to *j*: -Σ_i_Σ_j_p_ij_log(p_ij_), where p_ij_ is the probability that position *i* will transition to symbol *j*. *U*(random) is equal to the first-order entropy of a fully random transition matrix R_i,j_ (excluding where *i* = *j*).

#### Procedure

Participation consisted of three phases: (a) the training sequence, (b) a recall test, and (c) a report of explicit knowledge. Once participants opted to perform our task, they were forwarded to the Flash interface in their browser. Upon entry, the experimental software randomly assigned participants to a training stimulus sequence (each participant just saw one sequence). Participants were instructed to click the dots as fast as possible, because the interface was administering a RT task. The mouse cursor was continually tracked for the 48-position training stimuli. Circles appeared only one at a time, with a 750-ms inter-stimulus interval between them. This provided ample time for participants to initiate a predictive mouse movement. Following these responses, participants were prompted to produce a 24-position sequence from memory that matched what they had seen during training (akin to the “inclusion” task that likely marshals both implicit and explicit knowledge of the sequences; cf. Destrebecqz & Cleeremans, 2001). We tracked the circle positions that were clicked during this recall test. The four circle positions were all on the screen for this part of the task. Finally, participants rated how patterned they felt the 48-element sequence was by clicking on a continuous scale between *not patterned to completely patterned with somewhat patterned* in between.

#### Measures and analyses

We continually tracked the computer-mouse x,y-pixel coordinates during training. For each position appearance (of 48), we calculated two main measures on which our analyses are based: (a) initial distance (in pixels) to the next position and (b) initial distance (in pixels) from the previous position. For (a), we computed the pixel distance to the next target just before it appeared (at the 750-ms mark), which we call *initial distance to next*. If participants are making a perfect predictive movement, the initial distance will be 0 (i.e., right on top of the next circle position). A reactive behavior, by contrast, will have a larger initial distance, either near the previous target (“waiting”) or near the center (“readying”). However, for (b), a movement may be predictive but simply incorrect. We therefore also calculated the maximum horizontal/vertical distance from the previous target (at the 750-ms mark), which we call *initial distance from previous*. If a movement is predictive at all, it will have a large distance from previous, regardless of whether it is correct or not (see Figure 1, Panel B). As we describe further in the analysis below, these two measures provide windows onto predictive wagers of participants, and capture when participants are willing to invest mouse-cursor movements in a *particular* predicted stimulus. Any other trial, when the cursor does not make a large movement towards a particular stimulus, we term a *reactive trial*. Hypothetical illustrations of these trajectories are provided in Figure 1 (Panel C).

We also calculated the regularity of the test recall sequence, and its similarity to the corresponding training sequence that a participant saw. This is detailed below.

### Results

#### Analysis of reaction times

In an initial analysis, we simply tested whether our data indeed reflected traditional reaction-time facilitation across levels of sequence regularity (*G* score). In most statistical learning experiments, a control round using random sequences is used, to see whether RT or other measures are affected by the sudden change in the statistical structure of event sequences. Here, sequences of different *G* serve as relative, between-subject comparisons. We used a linear mixed-effects model with Subject as a random factor (Baayen, Davidson, & Bates, 2008), and Sequence Regularity *G* (0-1), Trial (1-48), and their interaction, as continuous fixed factors. *G* strongly predicted lower RT in this model, with each successive .1 increase in regularity leading to, on average, approximately 25 ms of facilitation, *F*(1, 139) = 42.5, *p* < .0001. There was also a significant effect of trial, with RT dropping by about 2 ms per trial, *F*(1, 6350) = 303.1, *p* < .0001. Importantly, these two factors interact, with high *G* sequences dropping *more* over trials than low *G* sequences, *F*(1, 6350) = 132.8, *p* < .0001. We therefore conclude that by a traditional analysis of RTs, we are able to show the same kind of results as found in previous work. The question, however, is whether these RT facilitation patterns can be accounted for by predictive movements. If so, then facilitation will arise from predictive mouse-cursor movements that get closer and closer to where the next trial stimulus will appear, even before the stimulus appears on the screen. We explore this possibility in the next two analyses.

#### Correct predictive movements

**Figure 2.**
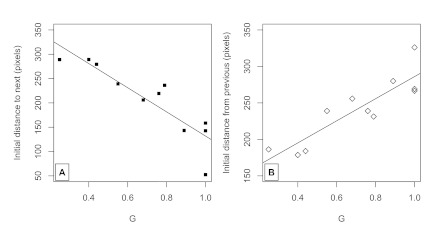
Panel A. Distance (in pixels and as a function of sequence regularity, *G*) to next stimuli after 750-ms lapse between disappearance of previous stimuli and onset of next stimuli. Panel B. Distance from previous stimuli after 750-ms lapse.

The overall extent to which participants moved the mouse cursor towards the next target prior to the target’s appearance was strongly related to the regularity of the grammar. This is shown in Panel A of Figure 2. Using the same model as described above, *G* highly significantly predicted initial distance to next. Each .1 increase in *G* on average led to about a 25-pixel closer initial position to the next target, *F*(1, 139) = 125.7, *p* < .0001. In general, each subsequent trial reduced initial position by about 1 pixel, *F*(1, 6351) = 174.2, *p* < .0001, but this depended upon *G*, indicated by a significant interaction term, *F*(1, 6351) = 161.2, *p* < .0001. In other words, high-*G* values (i.e., greater regularity) had a larger drop in initial position across trials compared to sequences with low-*G* values.

#### Predictive movements

We examined whether participants moved away from the previous target position using initial distance from previous. As described in the Measures and Analyses section above, if participants do not move at all, or only move towards the center, then this would be a “reactive” trial, because participants are only preparing for the next target to appear. However, if participants move away by a certain pixel distance, then they are likely moving the mouse cursor towards another target (either correctly or incorrectly). Such a trial would be a predictive one. Figure 2 (Panel B) shows the increase in initial distance from previous across sequence regularities. In short, participants become more predictive in the high-G sequences than low-*G*sequences. All the same effects hold in the same mixed-effects model (*ps* < .0001; we excluded the first trial from these analyses, as there is no previous trial to obtain such a measure). As suggested by the previous analysis, and in general, the extent to which predictive behaviors occurred over trials depended upon the predictability of the sequence itself.

#### Reactive-predictive strategies

**Figure 3.**
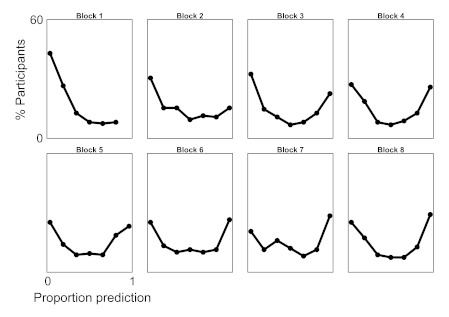
The percentage of participants (from all 11 *G*-score sequences) that exhibit reactive (proportion prediction = 0) or predictive (proportion prediction = 1) response modes across 48-position sequences divided into eight blocks. (Note that Block 1 shows 6 bins because Block 7 contained no participants. This 0 was included in the analysis, however.)

Simply averaging over participants (as in Figure 2) does not reveal a stark bimodality in predictive tendencies that we observed in our participants. To showcase this bimodality, we used the previous dependent measure (distance away from previous trial) and conducted distribution analyses. In the 48-position trials, any individual trial was deemed “predictive” when the initial distance from previous was 275 pixels or greater (indicating substantial movement away, likely to another target; see Panel B of Figure 1). We calculated the proportion of trials that were predictive in six-trial blocks (giving eight blocks). For any given block for each subject, a proportion score is obtained, lying between 0 and 1, representing the extent to which that block was predictive. A score of 1 on this proportion would indicate that all trials of these 6 were predictive. A score of 0 would indicate only reaction: Participants stayed closer to their initial position prior to the next trial. For each block, 1 to 8, a distribution of 143 scores is obtained (see Figure 3).

Each of these distributions is different from an assumed histogram of a uniform distribution of prediction proportions, χ(6)s > 30, *ps* < .0001. In the first block, participants are primarily reactive. Gradually, participants exhibit a sharp bimodal distribution in the final block. In inspecting the same histograms but for individual sequences, the distribution is as one would predict from the aforementioned analyses: High-*G*sequences have participants that transition to fully predictive trials; low-*G*sequences have participants that mostly remain reactive; bimodality holds approximately in the intermediate sequences.

#### Prediction occurs before correct prediction

**Figure 4.**
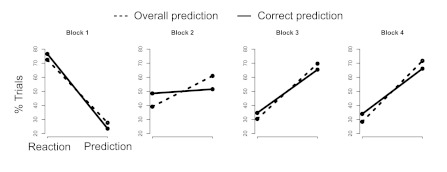
The distribution of trials in correct predictions (black line) and any predictive movement at all (dotted line). As seen in Block 2, overall prediction represents a larger proportion of the trials (approximately 70%) than correct prediction by itself (50%). In other blocks, prediction and correct prediction overlap closely.

We compared initial distance to next (correct prediction) and initial distance from previous (overall predictive movements) across blocks. If prediction occurs before knowledge, then overall prediction should be significantly higher at a crucial period as prediction emerges. Figure 4 shows the first four blocks of the three perfectly regular sequences, for which prediction was stable and frequent in participants. At Block 2, trials tended to be more predictive overall rather than simply correctly predictive. We generated a score from 0 to 6 for each block for each subject, computed by subtracting the number of correct predictions (using a conservative 100-pixel threshold) from the number of overall predictions by initial distance from previous (using the same 275-pixel threshold). In Blocks 1, 3, and 4 this score did not differ significantly from 0, as expected from Figure 4, *ts* < 1.9, *ps* = .10, .09, and .07, respectively. However, in Block 2, this score is substantially positive across these participants, *t*(39) = 5.1, *p* < .0001. This indicates that at about Block 2, there is more prediction in general than just correct prediction. In short, prediction generally appears to occur prior to total correct knowledge about the sequences.

#### Do the participants recall correctly?

**Figure 5.**
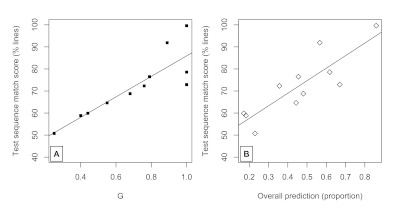
Panel A. Test sequence match score (%) as a function of *G*, with means grouped by stimulus condition. Panel B. Using the same stimulus list means, the match score as a function of predictiveness.

We calculated the grammatical regularity of the participants’ testing output of 24 clicks using the same statistic as in Jamieson and Mewhort (2009). In the expected direction, there is a strong relationship between the *G*-score regularity of the training sequence and the testing recall, *r* = .42, *p* < .0001. We also found that the testing sequences more closely matched the original training sequences in the same direction, *r* = .48, *p* < .0001. This matching score was generated using a sequence-alignment method known as *cross-recurrence* (see Dale & Spivey, 2005), where a percentage score reflects the overall match between the original and testing sequences by calculating the percentage of position sequences that are the same (similar to Levenshtein distance). In addition, we tested the relationship between how predictive a participant is in the final two blocks of the experiment (12 trials, using distance from previous with 275-pixel threshold), and the matching score, controlling for the training sequence *G*-scores (included as a covariate in a linear multiple-regression model). In the total model, training sequence *G*-score is a significant predictor (*p* < .01), but amount of prediction also strongly relates to the matching score (*p* < .005; multiple-*R*^2^ = .29, *p* < .0001). The relationship between matching score, and *G*, and predictiveness, is shown in Figure 5.

The same finding held for the relationship between prediction and test recall *G*-score (for prediction: *p* < .0001, multiple-*R*^2^ = .41, *p* < .0001). Interestingly, when factoring in how predictive participants are, training sequence *G*-score was no longer a significant predictor of the 24-item test recall *G*-score (*p* = .9). This suggests that the regularity of a participant’s memories for the sequences is somehow dependent upon their tendencies to actively predict the positions. Therefore, when controlling for the training sequence’s *G*-score, the tendency of participants to be predictive relates significantly to their performance on the test in both the free-response regularity and match to the training.

#### Explicit awareness correlates with all measures

**Figure 6.**
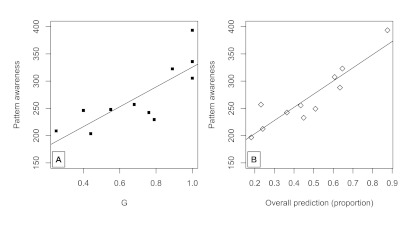
Panel A. Pattern awareness, a continuous-scale score from 100 to 400 (based on a clicked icon on the computer screen) as a function of *G*, means grouped by stimulus list. Panel B. Pattern awareness score as a function of overall predictiveness proportion, means grouped by stimulus list condition.

Participants who deemed the sequences to be more patterned tended to be the ones who had training sequences of higher *G*, *r* = .41, p < .0001. Higher explicit awareness of pattern related to greater test match to the training sequences, *r* = .47, *p* < .0001, and greater predictive behavior in the final 12 trials, *r* = .51, *p* < .0001. We ran a separate regression analysis to test for the relationship between prediction and explicit awareness while controlling for other variables, because prediction on the last 12 trials related significantly to these as well. First, we used the *G* score of a training sequence to predict perception of explicit awareness, and saved the residuals. When the *G*score was factored out of explicit awareness in this way, predictive tendencies still significantly accounted for what was left over in those residuals, *r* = .27, *p* = .005. The reverse is not true: Once prediction behavior is taken out of explicit awareness, *G* score is no longer significantly related to these residuals, *r* = .08, *p* = .4, suggesting that prediction mediates between a sequence’s regularity and explicit awareness. We chose to use participants’ test sequence generation’s match to training as an additional measure of explicit awareness, and both prediction and test recall correlated with explicit awareness, even when controlling for each other, *r*s > .25, *p*s < .005. The relationship between awareness score, and *G*, and predictiveness, is shown in Figure 6.

#### Is there really implicit learning at all?

It is important to reiterate that the current experiment is a very short one, using brief exposure on a simple sequence of four positions. Nevertheless, we sought to test whether there was any sign of implicit learning. As noted above, most statistical learning experiments use a control round with random (or different) sequences to see whether RT or other measures are increased by the change in the structure of sequences. Again, sequences of different G serve as relative, between-subject comparisons. If there is any implicit learning then participants who have low pattern awareness should nevertheless show improvement in RT across trials, moderated by the relative level of regularity in the sequence, as captured by *G*. We chose a subset of our participants who reported lower awareness of pattern (below but not equal to “somewhat patterned,” *n* = 25), and ran a linear mixed-effects regression with Subject as a random factor, and *G*, and Trial (1-48) as fixed factors, and included an interaction term. *G*was not alone significant, *F*(1, 29) = 2.1, *p* = .16, though trial was, *F*(1, 1404) = 19.9, *p* < .001. *G*and Trial interacted significantly, *F*(1, 1404) = 6.5, *p* = .01. The pattern of this interaction was as expected: The higher *G*sequences induced more learning across trials relative to lower *G*sequences. These participants reported low awareness of sequence pattern, and nevertheless showed modulated RTs relative to the regularity of the structure they received. This is at least suggestive of implicit learning in this brief exposure. As expected from previous analyses above, these participants also have significantly less predictive behavior in the final 12 trials than the remainder (*n* = 99) of the participants, *p* < .05.

### Discussion

These extensive analyses of our data offer some basic ideas about prediction and statistical learning, and their relationship to the implicit/explicit divide. First, prediction in the form of a “behavioral wager” tends to rapidly emerge as participants detect structure in the sequence. It is a stable strategy that does not seem to happen as a *result of learning*, but instead seems to *occur in conjunction with* early moments of learning. The results showing that incorrect prediction occurs as participants are transitioning into this predictive “mode” supports this. Below we further consider a “two system” hypothesis related to this: When an implicit learning process extracts sufficient structure, the cognitive system can “seek out” a forthcoming stimulus, thus producing an error signal, and kick starting explicit awareness and learning.

Second, explicit awareness measures (recall memory and awareness report) both correlate strongly with predictive behavior, even when controlling for sequence regularity. Thus, explicit knowledge appears to co-occur with “wagers” that participants are willing to invest in when they become aware of some structure. It may be that a particular kind of prediction (for a particular stimulus) is related to processes that unfold under conditions of conscious awareness.

As a final note, we observed that participants who did not wager often adopted the “centering” strategy that Duran and Dale (2009) found in their spatial statistical learning experiment. Participants anticipated the next stimulus, which occurred at one of three other locations, by positioning the mouse cursor at an optimal location, equidistant from the next stimulus (i.e., in the center). As we discuss further below, this may not be best described as *reactive*, as we have categorized it in our analysis, but rather an optimal anticipatory positioning close to possible future stimuli. Even participants with low pattern awareness engaged in this form of behavior.

## General Discussion

Admittedly, we designed a very simple task, and used it to explore *initial* response tendencies. Results thus reflect the processing of short-term event sequences that may be routinely faced by cognitive systems during daily activities (e.g., observing or producing brief structured action sequences; Botvinick & Plaut, 2004). Our experiment simplified this ecological context, and exposed participants to a single stream of visuospatial information. Certainly, the experiment is not of the same scope of traditional statistical learning and of SRT tasks, which use more complex sequences over many blocks of training. In that respect, what we are revealing is the very beginning of the learning system’s behavior, using computer-mouse trajectories to unveil the “microstructure” of this initial processing.

Results do suggest that the cognitive system, at least with respect to the manual motor system, is not constantly predicting the next particular stimulus event, especially in random environments. Instead, it adapts a readiness to respond, which may transition sharply into prediction once some regularity appears to be present. From here, prediction permits the generation of error (Rescorla & Wagner, 1972; Sutton & Barto, 1998), whereas simple reaction does not. From basic learning theory (Miller, Barnet, & Grahame, 1995), to more complex computational approaches of the past couple of decades (e.g., Elman, 1990), prediction is a wager the outcome of which leads to adjusted future expectations. It may be from such contingencies that deeply entrenched learning ensues.

Though our experimental findings appear robust, there are clear limitations to the current approach, which we hope to overcome in future investigations. Our simple design was deliberately short, seeking to observe and relate prediction in stimuli that are only brief. The optimal simultaneous “mixed strategy” (for middle *G*-scores), in which participants might both wager/center in predictable/unpredictable positions, is not observed but may emerge after extended training. Another issue, mentioned above in our experimental discussion, is that our participants often engaged in a consistent “centering” strategy. This is not mere reaction, as one would call the “wait and respond” strategy; yet it is not overt prediction either because participants are placing the cursor in an optimal position to react. Future work may seek to identify perhaps diverse response strategies that have various aspects of optimality depending on the task structure and instructions at hand, and could identify individual differences in this capacity to predict, either as an explicit or implicit strategy, which may relate to tendencies in other tasks (e.g., Proulx & Heine, 2009). In addition, our aggregate measures of memory may have underestimated the amount of explicit learning achieved during the task. It may be that explicit knowledge reported during the test is simply organized around particular sub-sequences (e.g., “4321”). Given the small number of participants per sequence, a statistical test of this hypothesis cannot be conducted here. Indeed, future work may demonstrate that computer-mouse movements form organized hierarchical units of predictive patterns, as suggested in work that models the organization of action sequences (e.g., Botvinick & Plaut, 2004). This would also bear on models that hypothesize different unitization of emerging statistical knowledge (e.g., Perruchet & Vinter, 1998).

We also focused on manual prediction tendencies, and did not look at oculomotor prediction. Previous work has shown the eyes to be widely predictive and the extent of this predictiveness to be related to explicit learning (D. J. Marcus et al., 2006), suggesting reactive-predictive strategies in oculomotor control could function distinctively (see also Land & Furneaux, 1997; Shelhamer & Joiner, 2003). Despite this potentially distinctive functioning, it is highly likely the unfolding of oculomotor and manual dynamics are coupled in natural contexts (Ballard, Hayhoe, & Pelz, 1995), and the relationship between their predictiveness could be explored with simple experiments like the one presented here. Indeed, the presence (or absence) of action contingencies is something that researchers in implicit statistical learning have debated (e.g., Heyes & Foster, 2002; Mayr, 1996; Willingham, 1999). The role of predictive processes is considered by many as central to this coupling between perception, action, and the environment. We feel our experiment could fruitfully connect prediction, statistical learning, and awareness of that learning in a single paradigm, and our results are suggestive of rich underlying relationships. We discuss this next.

### Implicit/explicit divide: Two systems?

We should preface our discussion here with an important note: Our results are correlational in nature. Prediction and awareness of a pattern are correlated in our task, as observed in past research (D. J. Marcus et al., 2006; Willingham, Nissen, & Bullemer, 1989). Yet, this awareness does not necessarily mean that a complete, explicit knowledge of the exact sequencing has been formed. Controlling for explicit knowledge by factoring out test sequence match, we still find that prediction occurs, suggesting that predictive behavior mediates the transition between a vague awareness of a pattern and the full-blown knowledge of that pattern (to which D. J. Marcus et al., 2006, also attest). Thus, explicit wagers of the kind we observe emerged rapidly in high *G*sequences, showcasing errors of prediction earlier in learning, and then settling into a stable strategy.

One subtle aspect of the debate in statistical learning in the past decade has been the extent to which it is taken to be anticipatory in nature (D. J. Marcus et al., 2006; Nissen & Bullemer, 1987; Schvaneveldt & Gomez, 1998; Stadler, 1989), or may be based on associative processes that need not always involve forward-looking mechanisms (Heyes & Foster, 2002; Jones & Pashler, 2007; Mayr, 1996). In fact, models used to capture this behavior have both properties as well, from local associative traces to more predictive processes (see Cleeremans & Dienes, 2008, for an elegant review of models). A tentative conjecture from our approach is that, as learners become acquainted with a sequential environment, their strategies may change depending on the regularity of that environment, and extent of exposure.

These strategies could reveal distinct underlying systems at work during learning. For example, in a study by Schvaneveldt and Gomez (1998), it was observed that single- versus dual-task learning contexts may induce different sorts of processes, with differing capacities to transfer that learning. They found in particular that knowledge gained in single-task learning was not easily transferred to a dual-task context when participants switched. Dual-task learning, however, induced knowledge that was transferrable to a new single-task context. As another example, previous researchers have debated the presence of two systems for learning, one based on attention to the material and the other not requiring attention (e.g., Curran & Keele, 1993), or whether just one system can account for such data (e.g., Frensch, Lin, & Buchner, 1998). Theoretical debate in implicit learning has often been geared towards identification of the subsystems involved, their properties, whether they operate alone or in parallel, and whether they produce abstract or concrete, or rule-based or statistical, knowledge (for reviews, see e.g., Cleeremans, Destrebecqz, & Boyer, 1998; Clegg, 2005; Conway & Christiansen, 2006; Curran & Keele, 1993; Frensch et al., 1998; Jamieson & Mewhort, 2009; Keele, Ivry, Mayr, Hazeltine, & Heuer, 2003; Kirkham, Slemmer, & Johnson, 2002; G. F. Marcus, Vijayan, Bandi Rao, & Vishton, 1999; Perruchet & Pacton, 2006; Reber, 1989; Seidenberg, 1999; Willingham & Goedert-Eschmann, 1999).

One may be tempted therefore to situate our results in this general trend to identify subsystems for learning. An implicit learning system may work to associate spatial positions over time, which facilitates spatial processing as they appear in sequence (debate also concerns whether there are subsystems here, regarding perceptual, attentional, and response-based bases; and recently there is suggestion that it could even exhibit modality specificity, cf. Conway & Christiansen, 2005). As sufficient structure is extracted by this general system, an “explicit” one kicks in, and participants begin to make explicit behavioral wagers about the next stimulus location. Indeed, there exists a prominent and related theory of cognitive control that also proposes two underlying modes of proactive and reactive operation (Braver, Gray, & Burgess, 2007). Yet we agree with Cleeremans and Dienes (2008) that identification of separate subsystems does not *by itself* count as an explanation of any data, and besides, as suggested, for example, in discussion by Kirkham, Slemmer, and Johnson (2002), what we observed could be multiple behavioral strategies emerging from a single general-purpose learning system. In fact, the modeling work of Destrebecqz and Cleeremans (2003) includes both an associative and predictive component that could capture both patterns we observe here. It may be (as we discuss below), that participants can simply verbalize this single system’s operation in some contexts, such as when prediction is possible, versus others, such as when only equidistant positioning (“centering”) is possible. Finally, even if we were to propose such a two-system explanation, further research is needed to judge whether, in our particular task, the associative, implicit system is still functioning even when overt behavior is overwhelmingly predictive (e.g., Willingham & Goedert-Eschmann, 1999).

Whatever the architectural description, there are clearly two strategies or “modes” observed in our behavioral data. And what is new in our observations is that these modes can rapidly transition from one into the other, from reactive practices into tentative prediction, and then to wholesale wagering that gives way to consistently correct predictions. And despite our task’s simplicity, we were able to go beyond previous research, in which such modes of learning or operating in sequential tasks are shielded by the wonderfully easy to acquire yet aggregate measure of RT; this can mask any interesting microstructure of unfolding statistical learning. Very elegant experiments can be designed to capture strategies using this measure (e.g., error and probabilistic sequences; Schvaneveldt & Gomez, 1998), by focusing on the RT distribution itself (e.g., very-short RTs; Willingham et al., 1989), or simply by inducing prediction during training or testing (e.g., Destrebecqz & Cleeremans, 2005); but in our design, the dynamics of prediction during learning are unveiled naturally and more transparently. Future work could identify the modes of operating, and the principles that guide them, by exploiting the behavioral measures we used here (cf. Duran & Dale, 2009; Moisello et al., 2009; D. J. Marcus et al., 2006).

### Implicit/explicit divide: Prediction and awareness

Previous studies have also found that indices of prediction correlate with explicit knowledge of the presence of regularity of a sequence (D. J. Marcus et al., 2006; Willingham et al., 1989), and our results replicate this connection. What is especially novel in our results, the study’s limitations notwithstanding, is that the time course of the onset of this prediction can be captured. The data reported here suggest that participants rapidly initiate prediction as a strategy that accompanies the acquisition of knowledge, rather than being a direct consequence of it (cf. D. J. Marcus et al., 2006). As mentioned at the outset of this paper, many researchers have identified prediction as a central process underlying much of perception, cognition, and action. It has been implicated in high-level cognitive processes, such as explicit awareness of causal agency (e.g., temporal relations in action-effect for judgment of authorship; Wegner, 2003) and self-awareness (see Jeannerod, 2006, for a review of relevant evidence), and even at lower levels, as a foundational process of action and perception, since prominent theories of action control still propose that the cognitive system predicts the consequences of actions (e.g., Hommel, 2009). Yet the fact that prediction seems to relate to implicit/explicit knowledge in statistical learning has not gained much theoretical attention.

One recent account that may explain why explicit prediction of the kind measured here - which we have often referred to as *wagers* - is the theory of Morsella (2005), which explains phenomenal states as emerging in cross-modal and integrative contexts that converge to control body plans. When diverse information (e.g., from multiple modalities) converges on action plans, the states that accompany such a condition have phenomenal properties that may function to bind these diverse information sources into a likeness of experience that we typically call *consciousness, awareness*, and so on. When wagering prediction occurs, it may reflect a convergence of information from prior perception and action experiences that, in Morsella’s (2005) terms, interfere with ongoing body plans, and phenomenal states reflect the cognitive system’s integration or binding of these experiences in order to maintain skeletomotor control. In this sense, the strategy of explicit, stimulus-specific prediction must draw the motor system away from other possible association and drive the system toward a particular location; implicit associative processes may involve parallel processes that compete more “benignly” and do not require phenomenal binding processes to anchor them. This proposed distinction can only be treated as gradient and approximate, however, because there is evidence for *implicit* predictions in statistical learning in other work (e.g., Turk-Browne, Scholl, Johnson, & Chun, 2010). It turns out that these more implicit process do seem to relate to visual prediction (e.g., Bar, 2009), where underlying associative representations may be employed for relatively implicit, rapid expectations; whereas in ours and previous studies, explicit awareness accompanies motoric manifestations of prediction, as perhaps Morsella (2005) would hypothesize. Future research may reveal that prediction bears in different ways on perceptual and response-based implicit/explicit learning.

The foregoing discussion is not meant to argue that explicit knowledge is required for learning, and indeed our data are suggestive of the early stages of implicit learning in very simple sequences. We also do not wish to take up the notion that implicit/explicit learning systems are architecturally distinct; there are other perspectives on these issues that do not necessarily require complete separation of processes or their resulting knowledge in order to account for experimental data, whether one rejects any such dissociation (e.g., Perruchet & Amorim, 1992), or embraces a more gradient perspective on implicit/explicit knowledge (Cleeremans & Jiménez, 2002). Regardless of one’s theoretical stance on architectural separation or distinct functioning modes of a single learning system, the surface behavior that is exhibited in this simple SRT task suggests that there are indeed different modes or strategies that emerge during learning. We would argue that the experimental paradigm we have presented may help mitigate these kinds of theoretical debates.

This discussion is, of course, purely speculative with regard to the current experiment, and there is extensive discussion about explicit “conscious” awareness of such things as the actions that unfold during everyday tasks, of why such actions have taken place, and of the learning that may take place during these actions (Cleeremans et al., 1998; Cleeremans & Jiménez, 2002; Cohen & Schooler, 1997; Haggard, Clark, & Kalogeras, 2002; Hurley, 2002; Jeannerod, 2006; Reber, 1992; Sarrazin, Cleeremans, & Haggard, 2008; Van Orden & Holden, 2002; Wegner, 2003), that we do not have space to consider here. The excitement in the field regarding prediction, and the continued interest in consciousness as an outstanding puzzle of the cognitive sciences, may be a point of synergy between basic research on predictive cognition and philosophical discussion of explicit awareness of learning that takes place during everyday life. The field thus requires the development of new techniques to tap predictive tendencies in laboratory contexts, and relate these tendencies to implicit or explicit processes that underlie statistical learning. We hope readers find the paradigm and experiment we offer here as a promising means by which this can be pursued to bridge the divide.
